# A Virtual Self-Management Intervention for Adolescents With Juvenile Idiopathic Arthritis: Protocol for the VISTA-JIA Randomized Controlled Trial

**DOI:** 10.2196/69539

**Published:** 2025-06-27

**Authors:** Jessica Booth, Kathryn A Birnie, Kelsey Chomistek, Maria Santana, Jennifer N Stinson, Babatope O Adebiyi, Julia Brooks, Jaime Guzman, Robin Hellweg, Lily SH Lim, Dax Rumsey, Brian M Feldman, Jamie Tagseth, Jennifer Wilson, Heinrike Schmeling

**Affiliations:** 1 Section of Rheumatology, Department of Pediatrics Cumming School of Medicine University of Calgary Calgary, AB Canada; 2 Department of Community Health Sciences, Department of Anesthesiology, Perioperative, and Pain Medicine University of Calgary Calgary, AB Canada; 3 University of British Columbia Vancouver, BC Canada; 4 Department of Pediatrics Cumming School of Medicine and Department of Community Health Sciences University of Calgary Calgary, AB Canada; 5 Research Institute, The Hospital for Sick Children and the Lawrence S. Bloomberg Faculty of Nursing University of Toronto Toronto, ON Canada; 6 Alberta Children’s Hospital Calgary, AB Canada; 7 Division of Rheumatology, Department of Pediatrics BC Children’s Hospital University of British Columbia Vancouver, BC Canada; 8 Children’s Hospital Research Institute of Manitoba Department of Paediatrics University of Manitoba Winnipeg, MB Canada; 9 Section of Rheumatology Department of Pediatrics University of Alberta Edmonton, AB Canada; 10 Division of Rheumatology The Hospital for Sick Children University of Toronto Toronto, ON Canada; 11 Cassie and Friends, Canada Vancouver, BC Canada; 12 Alberta Children’s Hospital and McCaig Research Institutes Cumming School of Medicine University of Calgary Calgary, AB Canada

**Keywords:** juvenile idiopathic arthritis, self-management program, randomized controlled trial, videoconference, virtual intervention, peer support

## Abstract

**Background:**

Needs assessments in patients with juvenile idiopathic arthritis (JIA) have revealed a need for disease information, self-management skills, and peer support. We previously developed and tested the acceptability of an in-person and videoconference-based self-management program (SMP) to address these needs.

**Objective:**

The aim of this pilot randomized controlled trial (RCT; the VISTA-JIA trial) is to evaluate the feasibility and preliminary effectiveness of a virtual group-based SMP for adolescents with JIA in comparison to a waitlist control group.

**Methods:**

A total of 100 participants with confirmed JIA (aged 12-17 years) will be recruited from 5 Canadian pediatric rheumatology centers and randomized 1:1 to the intervention or waitlist control groups. Adolescents in the intervention group will receive the virtual SMP. Those randomized to the control group will receive standard of care alone and will later be eligible for the SMP. The SMP includes JIA disease education, self-management strategies, and peer support. Four 60- to 90-minute sessions will be conducted over 8 weeks with a group size of 4-6 participants. The primary feasibility outcome will be adherence to the SMP (defined as completion of all 4 sessions by at least 80% of participants). Other secondary feasibility outcomes will include recruitment and withdrawal rates, the proportion of completed questionnaires, engagement and satisfaction with the SMP measured through a semistructured virtual interview, and intervention fidelity (consistent content and technology delivery). Secondary preliminary effectiveness outcomes will be assessed by completing 5 validated questionnaires at pre- and postprogram time points: (1) the Medical Issues, Exercise, Pain, and Social Support Questionnaire to assess perceived ability to manage JIA (self-management); (2) the Children’s Arthritis Self-Efficacy Scale to assess self-efficacy; (3) the Pediatric Quality of Life Inventory 3.0 Rheumatology–Teen Module to assess health-related quality of life; (4) the PROMIS (Patient-Reported Outcomes Measurement Information System) Pediatric Pain Interference Scale to assess pain interference; and (5) Readiness for Adult Care in Rheumatology to assess transition readiness. Descriptive statistics and nonparametric tests will be used to analyze the data.

**Results:**

The study setup is complete at all centers, including training of the facilitators, revising and finalizing education sessions, participant’s handout guide, and fidelity checklist. Recruitment began in January 2024 and is expected to conclude by December 2025. Feasibility outcomes, including adherence and engagement, as well as preliminary effectiveness, will be analyzed post intervention.

**Conclusions:**

This is the first evidence-based, virtual, interactive, group-structured JIA SMP in Canada. This SMP will address needs for disease information, self-management skills, and peer support in adolescents with JIA. The results of this pilot study will inform a full-scale RCT focused on the efficacy and cost-effectiveness of the program with the goal of integration in routine clinical practice across Canada.

**Trial Registration:**

ClinicalTrials.gov NCT06184100; https://clinicaltrials.gov/study/NCT06184100

**International Registered Report Identifier (IRRID):**

DERR1-10.2196/69539

## Introduction

### Background

Juvenile idiopathic arthritis (JIA) is the most common childhood rheumatic disease with an estimated prevalence of 1 in 1000 children younger than 16 years [1]. Although treatments for JIA have improved over the last decade, many affected children experience continued disease activity and related morbidity, long-term disability, and an increased incidence of psychosocial complications as they enter adulthood [2]. Adolescence is a critical period of physical, cognitive, and psychosocial development as the youth develop skills, emotional regulation, and independence to manage changes in responsibilities and prepare to assume adult roles [3]. In adolescents with JIA, this period translates to a requirement for additional self-management skills (eg, medical decision-making and patient-provider communication) as their autonomy increases and as they assume greater responsibility for the management of their disease to prepare for transition into adulthood and adult health care. Unfortunately, 25%-75% of adolescents with JIA are lost to follow-up once transferred from pediatric to adult health care in Canada [4-8]. Needs assessments have revealed insufficient time to provide transition services, a lack of reliable JIA information accessible to adolescents and knowledge about linkages to community resources, need for additional self-management skills, and a gap in peer support in adolescents with JIA [9-13]. These gaps were found to be critically important to address within individual countries through distinctively customized plans and activities [4,14].

### Peer Support and Self-Management Programs

Self-management programs (SMPs) are proposed as effective ways to address the educational, self-management, and peer support needs for adolescents with JIA. Significant evidence from adult and pediatric medicine has indicated that SMPs result in improved health behavior, self-efficacy, and certain health status outcomes in persons with arthritis [15,16]. SMPs afford the opportunity to instantly react to participant feedback, tailor the amount and complexity of information to participants’ needs [17,18], and immediately address information that participants do not understand. Furthermore, SMPs in a small group setting allow for interaction and discussions among participants, which, in turn, builds social support [15]. Remotely delivered SMPs, in the form of videoconferencing, represent an evolving technology that can improve accessibility, especially for patients residing in rural or remote communities [19].

Previous needs assessments [12-14] have identified the need for virtual self-management interventions incorporating peer support and for standards of technology delivery and guidance for effective virtual communications in a group setting [20,21].

Our aim is to conduct a pilot multicenter randomized controlled trial (RCT) to evaluate the feasibility and preliminary effectiveness of a virtual group-based SMP for adolescents with JIA in comparison to a waitlist control group.

## Methods

### Study Design

**Figure 1:**
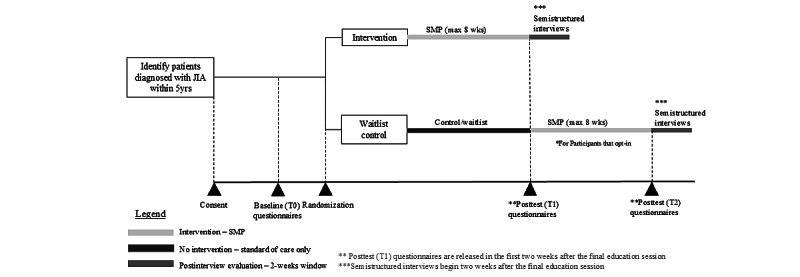
Trial schema.

A pilot RCT will be conducted to determine the feasibility and preliminary effectiveness of a virtual SMP intervention. Eligible participants will be randomized 1:1 to either the intervention group or a waitlist control group. Both groups will complete baseline measures (T0) as well as postprogram measures (T1). Participants enrolled into the intervention will undergo the SMP and be asked to participate in an optional postintervention interview. The waitlist control group will continue receiving standard of care during this program period. They will be given the option to complete the SMP afterwards, and if completed, be asked to complete postintervention questionnaires. They will also be asked to participate in the optional postintervention interview. The study is registered in ClinicalTrials.gov (NCT06184100). Figure 1 shows the trial schema, and Multimedia Appendix 1 shows the timeline. This study follows the SPIRIT (Standard Protocol Items: Recommendations for Interventional Trials; checklist provided in Multimedia Appendix 2) guidelines.

### Participants

Participants will be recruited from 5 Canadian pediatric rheumatology centers across different provinces after obtaining local research ethics approval. Sociodemographic and disease-related characteristics including age, gender, disease subtype, date of diagnosis, and current or past treatment will be collected from health records for descriptive purposes at baseline.

### Inclusion Criteria

Participants will be considered eligible to participate if they meet the criteria described below:

1. Adolescents between the ages of 12 and 17 years.
2. Confirmed diagnosis within 5 years according to the International League of Associations for Rheumatology JIA classification criteria.
3. Followed up in one of the pediatric rheumatology clinics participating in the RCT.
4. Able to access the internet.
5. Willing and able to complete web-based measures.

### Exclusion Criteria

Individuals will be excluded from the RCT if they meet any of the following criteria:

1. Insufficient English reading and speaking skills.
2. Untreated psychiatric or comorbid disorders or major cognitive impairments leading to an inability to understand materials and participate in the SMP group activities.
3. Other chronic conditions such as other autoimmune disease, neurologic, orthopedic, and organ system disorder (eg, heart and kidney) that might influence outcome assessments.
4. Past participation in the last year or participating in another formal peer-support or self-management program.
5. Additionally, concomitant adjunct interventions and other involvement with peer support groups (contamination) will be recorded.

### Randomization, Recruitment, and Sample Size

CONSORT (Consolidated Standards of Reporting Trials; checklist provided in Multimedia Appendix 3) 2010 guidelines suggest a sample size calculation of 9% of the anticipated full-scale RCT for the intervention group in pilot studies [22,23]. However, to establish feasibility at several sites and get an estimate of a statistically significant SD to inform a full-scale multicenter trial, our target is to conduct 2 offerings of the program with 4-6 participants per group at each of the 5 sites to control for site-specific education. Eligible participants will be identified through a review of the patient database and will be contacted by research coordinators. Some participants will also be recruited during their clinic visits. They will receive detailed information about the study, including its objectives, procedures, and potential benefits. Interested participants will provide informed consent (or assent, with parental consent for minors). The study aims to recruit a total of 100 participants. Therefore, we will require 50 participants in the intervention group and 50 in the waitlist control group. This sample size was chosen to establish feasibility and inform a larger multicenter trial. Participants will be randomized 1:1 to either the intervention group or the waitlist control group using block randomization with variable block sizes. This randomization will be conducted using REDCap (Research Electronic Data Capture; Vanderbilt University), a secure web-based application for managing data. Randomization will occur before baseline measures are completed. Participants in the intervention groups will be from the same center to adapt the program to local health care systems and account for the variability of nonformal educational practices between centers. This approach will also facilitate an evaluation/subgroup analysis of outcome measures at each center.

### Intervention

The program consists of JIA disease education, self-management strategies, and peer support in a group-based setting. Four sessions, 60-90 minutes each, will be conducted over 8 weeks with a group size of 4-6 participants via videoconferencing platforms. Each session will include Microsoft PowerPoint–delivered presentations, interactive activities, and group discussions. The 4 sessions will focus on the following themes: Overview and Diagnosis of JIA, Daily Living and Exercise, Coping Strategies, and Medication and Lifestyle Management. To ensure intervention fidelity, sessions will be provided by the same designated health care professionals. All sessions will be video-recorded and uploaded to a secured research server to assess the group’s dynamic and ensure sessions are delivered as planned (fidelity). Young adults living with JIA from participating centers will be asked to sit in during these sessions to share their lived experiences and foster peer social support. Additionally, each site will include site-specific resources. After completing the intervention, participants will be asked to independently attend an optional semistructured interview to capture participant experiences of the intervention.

### Session Delivery and Training

The National Institute of Health’s (NIH’s) best-practice guidelines on intervention fidelity [24] will be used to develop and establish standards for virtual session delivery. This will incorporate the NIH’s framework principles: consistency in intervention content information and delivery and training the interventionist for delivering a consistent intervention. To achieve this, we will develop an interventional manual on the technology setup and troubleshooting, ground rules and videoconferencing etiquette with participants, how to maintain consistency in content delivery, techniques to engage participants and guide discussions, and the content of a checklist that will be used to assess intervention fidelity.

Second, we will provide training to the interventionists. Training sessions will occur prior to program implementation using our manual. Interim feedback will be provided after 2 and 6 sessions using fidelity checklists to maintain consistent delivery and continuous process improvement.

### Outcome Measures

#### Feasibility Outcomes

Primarily, feasibility will be measured through adherence with the SMP (defined as 80% completion). Other feasibility outcomes will include the following:

1. Recruitment and withdrawal rates (>80% recruitment rate and <80% withdrawal).
2. The proportion of completed questionnaires (defined as 80% when all measures are completed) at baseline and 8 weeks post randomization in both groups.
3. Satisfaction with the SMP as measured through questionnaires and a semistructured virtual interview with the participants following involvement in the SMP to capture the participants’ experience.
4. Intervention fidelity (defined as consistent content and technology delivery) will be assessed by fidelity checklists and qualitative analysis of recorded video sessions after 2 sessions, 4 sessions, and at completion of all sessions to ensure that sessions are consistently delivered and as planned.

#### Preliminary Effectiveness Measures

Participants of both the intervention and control groups will complete 5 validated and reliable patient-reported outcome measures on the web at baseline and post program. This includes the following:

- The Medical Issues, Exercise, Pain, and Social Support Questionnaire—a validated (eg, concurrent validity and construct validity) and reliable (eg, internal consistency) measure for perceived ability (eg, knowledge, skill, behavior, attitudes, and self-efficacy) to manage JIA [25].
- The Children’s Arthritis Self-Efficacy Scale—a validated (eg, concurrent validity and construct validity) and reliable (eg, internal consistency) measure for arthritis self-efficacy [26].
- The Pediatric Quality of Life Inventory 3.0 Rheumatology–Teen Module—a validated (eg, construct validity and concurrent validity) and reliable (eg, internal consistency) measure used for pediatric rheumatology–specific health-related quality of life in children aged 13-18 years [27].
- The PROMIS (Patient-Reported Outcomes Measurement Information System) Pediatric Pain Interference Scale—a validated (eg, concurrent validity and construct validity) and reliable (eg, internal consistency) measure for pain behavior in children aged 8-17 years [28].
- Readiness for Adult Care in Rheumatology—a validated (eg, concurrent validity and construct validity) and reliable (eg, internal consistency) measure to assess transition readiness in adolescents with JIA [29].

### Ethical Considerations

This study was approved by the Conjoint Health Research Ethics Board of the University of Calgary (REB22-1820), Edmonton (pSite00000101), Vancouver (H24-02469), SickKids Research Ethics Board (1000080939), and the Health Research ethics Board of the University of Manitoba (HS26016{H2023:168}). Informed consent will be obtained from all participants in accordance with the tenets of the Declaration of Helsinki, with parental consent required for minors. Participants will be informed of their right to opt out at any time. Data collected will be anonymized to ensure privacy and confidentiality, with protective measures in place to safeguard the participants’ information. Compensation for participation will be provided in the form of gift cards valued at CAD $50 (US $36.51) per participant. No identification of individual participants will be possible in any images or supplementary material included in the manuscript.

### Data Analysis and Statistical Methods

#### Sociodemographic Characteristics

Descriptive statistics (mean, SD, and IQR) will be used to analyze sociodemographic characteristics.

#### Data Analysis Plan

##### Feasibility Outcomes

The feasibility of the SMP will be analyzed using descriptive statistics (eg, mean, SD, and frequencies) based on (1) adherence with the SMP (defined as 80% when the participant completes 4 sessions over 8 weeks), (2) recruitment and withdrawal rates, and (3) the proportion of completed questionnaires at baseline and 4 weeks after the last session (defined as 80% when all measures completed).

This study will use qualitative thematic analysis [30,31] to analyze the data. The experiences, perceptions, and satisfaction measured through video-recorded semistructured individual interviews will be transcribed verbatim and entered to a data analysis software (NVivo version 12.0; Lumivero) [32] for analysis. This method involves iterative cycles of coding, interpreting, and reflecting on the data to identify patterns and themes that capture participants’ experiences and perceptions of SMP. Key nonverbal cues, terms, and phrases will be noted and underlined in the transcript and coded accordingly. All qualitative data will be read separately several times by the research assistant and at least 2 investigators to obtain an overall understanding of the data, generate initial codes, and develop themes based on research questions. The themes will be refined, defined, and supported by extracts from the transcript. Finally, the themes will be documented in a manuscript.

This study will ensure rigor and trustworthiness through credibility, transferability, dependability, confirmability, and reflexivity [33]. A detailed description of the study setup, participants, and data collection procedures will support transferability. Dependability will be ensured by documenting data collection, analysis, and interpretation. Two researchers will independently code transcripts and resolve discrepancies through consensus. A single interview schedule will guide all interviews, and member-checking will enhance credibility. A reflective journal will document research discussions as part of the audit trail. In addition, verbatim transcripts of participants’ responses will be included in the manuscript to ensure confirmability [33]. Using the same qualitative methods, we will evaluate intervention fidelity of the recorded video sessions. Scores from the developed fidelity checklist will be used to further support the evaluation of intervention fidelity. Positive checklist ratings (scale 0-10, where 10 is the most positive) will indicate an intervention that is delivered consistently and as planned.

##### Preliminary Effectiveness Outcomes

The quantitative data will be entered into SPSS [34]. Descriptive statistics will be used to analyze the patient-reported outcome measures with an ordinal Likert scale at baseline and immediately post intervention. In addition, nonparametric Wilcoxon signed rank tests will be used to assess if there is a significant difference between a pre- and posttest outcome. Nonparametric tests will be used due to the small sample size and a lack of normally distributed data. Changes in pre- and posttest outcomes will be analyzed over time using a repeated measures mixed linear model approach where participants will be included as the random effect and baseline scores, study site, and disease-related characteristics will be used as fixed factors or covariates.

##### Subgroup Analysis

To initiate preliminary exploration of interventional effectiveness by study site and/or patient characteristics (age, sex and gender, disease subtype, and disease severity) interactional statistical tests (such as linear regression models for continuous variables) will be used to detect if differences exist. This will inform future trial design (eg, which strata to include in randomization based on detected subgroup differences).

#### Missing Data Considerations

We will examine patterns of missing data, determine demographic and/or clinical characteristics that are related to missing data at study time points, and the potential impact on the primary findings. Patterns will be used to inform our use of an intention to treat and to determine if including a per protocol approach in the full-scale RCT will be appropriate.

#### Data Integration

To address the main research questions related to feasibility and primary effectiveness for scale-up, we will integrate qualitative and quantitative data using a convergent parallel design [35]. This method involves the simultaneous collection and separate analysis of both data types, followed by merging the results for comprehensive insights. Quantitative data from validated questionnaires will provide measurable outcomes on self-management, self-efficacy, health-related quality of life, pain interference, and transition readiness. Concurrently, qualitative data from semistructured virtual interviews will capture participants’ experiences, engagement, and satisfaction with the SMP. By comparing quantitative results with themes from the qualitative analysis, we will identify patterns, discrepancies, and complementary findings. This holistic approach ensures that both numerical trends and personal narratives are considered together, offering a robust understanding of the program’s feasibility and effectiveness, which will inform potential scale-up and integration into routine clinical practice.

#### Sex and Gender Considerations

Female sex is a biological risk factor for JIA. Gender is a complex construct encompassing individual social role, behavior, and identity. Gender may be influenced by or interact with multiple factors including age, pain, or symptom perception, and these interactions may impact adherence to and satisfaction with the SMP. We will, therefore, consider the impact of sex and gender on our outcomes as described above. For all participants, we will collect information on sex (at birth) as well as current gender identity to facilitate analysis and reporting by sex and gender separately where appropriate.

## Results

### Overview

Recruitment for the VISTA-JIA study began in January 2024 and is expected to conclude by December 2025. As of March 2025, a total of 40 participants have been successfully enrolled from 5 Canadian pediatric rheumatology centers. The recruitment process involved approaching eligible participants during clinic visits and providing detailed information about the study, including its objectives, procedures, and potential benefits. Informed consent (or assent, with parental consent for minors) was obtained from interested participants.

### Session Delivery and Training

We have developed and established standards for virtual session delivery based on the NIH’s best-practice guidelines on intervention fidelity. This incorporates the NIH’s framework principles: consistency in intervention content information and delivery and training the interventionist for delivering a consistent intervention. Training sessions for the interventionists have been conducted. One education intervention has been conducted and will be used to further improve the program.

## Discussion

### Anticipated Findings

This pilot RCT aims to evaluate the feasibility and preliminary effectiveness of a virtual SMP for adolescents with JIA. The anticipated main findings include high adherence rates (≥80%) to the SMP, positive engagement and satisfaction among participants, and preliminary improvements in self-management skills, self-efficacy, and transition readiness. Additionally, the study is expected to demonstrate the feasibility of conducting a virtual group-based intervention across multiple pediatric rheumatology centers in Canada.

The need for self-management interventions in JIA has been well-documented in previous research [12-14]. Prior studies have highlighted the challenges adolescents face in managing their disease, particularly during the transition to adult care [9-13], and the benefits of peer support in chronic disease management [15]. Previous in-person SMPs have shown effectiveness in improving self-efficacy and disease knowledge [15,16], but accessibility has remained a barrier, particularly for those in rural or underserved areas [19]. The VISTA-JIA study builds on this evidence by delivering a structured, interactive, and virtual SMP, leveraging videoconferencing technology to improve accessibility and scalability. This approach aligns with emerging trends in digital health and telemedicine, which have shown promise in other chronic disease management programs [19].

### Significance

This will be the first, to our knowledge, evidence-based self-management program to offer virtual self-management participation for adolescents with JIA. If proven feasible and acceptable, this SMP will be then tested in a larger RCT with enough participants and statistical power to demonstrate the effectiveness of the SMP across pediatric rheumatology centers in Canada. The program’s unique design to include both health care providers (HCPs) and peer participants within a group setting, provides an opportunity to (1) enhance and instill confidence in participants’ individual ability to self-manage skills under the guidance of experienced HCPs; (2) allow for participants to gain a better understanding of JIA through questions and input provided by their peers; (3) enhance their emotional management skills by learning through the shared challenges and emotional perspectives of participants within the group; (4) potentially foster a network of social support among the participants, which may prove beneficial beyond the scope of this project and their transition to adult care; and (5) streamline the need for HCPs’ educational practices and optimize for consistent dissemination of information with engaging and interactive activities. Additionally, assessing intervention fidelity will allow us to establish a standardized framework for content and technology delivery and will help to train future session facilitators. This will finally be applicable to broader audiences as our establishment of a standardized technology-delivery framework and guidance for effective virtual communications in a group setting can be applied to any virtually delivered intervention in health care.

### Strengths and Limitations

Integrating peer support into a virtual SMP provides a unique opportunity to foster social connections, which has been identified as a critical component of effective self-management. The structured group format promotes interaction and shared learning, which can further enhance self-efficacy and engagement. The use of a virtual platform is another major strength, as it improves accessibility for participants, particularly those in remote or underserved areas. This is particularly relevant for chronic disease management, where sustained engagement in self-care practices is essential. Furthermore, this study incorporates rigorous methodological approaches to ensure intervention fidelity, including standardized session delivery, comprehensive facilitator training, and session recordings for quality control. By implementing best practices in virtual intervention design, the study establishes a replicable model for future digital health interventions in pediatric chronic disease management.

This study may not be generalizable to the broader JIA population. However, to improve generalizability and adaptability, we have partnered with 5 centers across Canada that serve patient populations from heterogeneous backgrounds. This includes rural, urban, and ethnically diverse locations. As a web-reliant intervention, this trial is subject to some of the inherent vulnerabilities that accompany the use of internet-based platforms such as varied connectivity access and reliability among participants. To address this, we intend to plan for contingencies, such as sending participants recorded sessions and scheduling a follow-up call to address any questions or concerns.

### Future Directions

Findings from this pilot study will inform the design of a full-scale RCT to evaluate the program’s long-term effectiveness and cost-effectiveness. Future research should explore ways to optimize participant engagement, including the use of asynchronous learning modules to complement live sessions. Additionally, an evaluation of caregiver involvement in adolescent self-management may provide further insights into enhancing program impact.

### Dissemination Plan

This plan will involve the following:

1. Multiple partners (Cassie and Friends Society, Alberta Strategy for Patient Oriented Research SUPPORT Unit), collaborators (allied health professionals), and knowledge users (young adults with JIA) have been and will continue to be kept engaged in the development and evaluation of the SMP and involvement in all knowledge translation activities as members of the knowledge translation committee. Members will be updated on the study progress; their perspectives will be sought for interpretation of study findings and on ways to share knowledge through their respective organizations.
2. Findings will be shared with health care professionals via presentations at key national and international conferences, Canadian Alliance of Pediatric Rheumatology Investigators and Childhood Arthritis and Rheumatology Research Alliance meetings, web-based webinars, seminars and rounds at hospitals and universities, and publications in open access journals.
3. Findings will be shared with patients and consumer groups (1-page brochure, media releases [newspaper, magazines, and social media such as YouTube videos], posting on key websites [eg, hospitals, Cassie and Friends Society, and the Arthritis Society]), and online education sessions aimed at families through research team and partners.
4. The research team will support consumer representatives in translating study findings using consumer fact sheets to educate their health providers (“consumer detailing”). This strategy has been suggested as one of the most potent methods for changing practice and disseminating new knowledge.

### Conclusions

The findings from this research have the potential to inform the development of programs that equip adolescents with JIA with the necessary skills to manage their own care effectively. By fostering greater self-efficacy in managing their condition, we anticipate improved transitions from pediatric to adult care and enhanced long-term health outcomes for individuals with JIA. Furthermore, this study will contribute to the broader efforts to establish standardized approaches for virtual SMPs and educational interventions, with implications for the management of various pediatric chronic conditions. This pilot study will inform a larger RCT focused on the efficacy and cost-effectiveness of the program with the goal of integration in routine clinical practice.

## Data Availability

The datasets used during the current study are available from the corresponding author on reasonable request.

## Appendix

Multimedia Appendix 1 Peer reviewer report from Strategic Operating Grant (SOG), Integrated Scientific and Medical Advisory Committee, Arthritis Society Canada.

Multimedia Appendix 2 SPIRIT 2022 checklist.

Multimedia Appendix 3 CONSORT-eHEALTH checklist (V 1.6.1).

## Abbreviations

CONSORT: Consolidated Standards of Reporting Trials
HCP: health care provider
JIA: juvenile idiopathic arthritis
NIH: National Institutes of Health
PROMIS: Patient-Reported Outcomes Measurement Information System
RCT: randomized controlled trial
REDCap: Research Electronic Data Capture
SMP: self-management program
SPIRIT: Standard Protocol Items: Recommendations for Interventional Trials
