# Supplementary material for: A Virtual Self-Management Intervention for Adolescents With Juvenile Idiopathic Arthritis: Protocol for the VISTA-JIA Randomized Controlled Trial
Source: JMIR Res Protoc. 2025 Jun 27;14:e69539. doi: 10.2196/69539 (PMC12254703; doi:10.2196/69539)
Supplement: Multimedia Appendix 1 [file resprot_v14i1e69539_app1.pdf]

Application: 22-0000000212

Heinrike Schmeling - heinrike.schmeling@albertahealthservices.ca
Strategic Operating Grant (SOG)

Summary

ID: 22-0000000212
Last submitted: 11 Jan 2024 05:07 PM (EST)

Your Reviewer Feedback Report - Strategic Operating Grants Full Application

Completed - 19 Sep 2024

Reviewer Feedback Report - SOG Full Application

Your application has been evaluated by a peer review panel and considered by Arthritis Society Canada's Integrated Scientific and Medical Advisory Committee.

Any questions you may have regarding your application will be answered in this feedback report. We urge you to use this feedback in the constructive manner for which it was intended.

Please direct any questions to research@arthritis.ca

This report will be available until January 26, 2024. To download this report, select the ellipses on the upper right side of this form and select 'download'.

Reviewer Feedback Report

Applicant Name: Heinrike Schmeling
Application ID: 22-0000000212

Primary Reviewer Feedback

Scientific Merit Strengths

- Research strategy:
  - Background: prior work in assessing and implementing self management programs has been done in small samples (<50) and only two prior studies used an RCT design. Prior work has raised concerns about feasibility: low recruitment, poor adherence, and attrition. A videoconference group-based self-management program may overcome some limitations of prior studies. In addition, the larger sample size will allow preliminary assessment of efficacy outcomes.
  - Innovation: preparing adolescents (age 12-17) as they begin to transition into adulthood and adult heath care, where they will assume greater responsibility for managing their disease. Prior work has shown enormous lost-to-follow-up rates (25-75%) as children with JIA transfer from pediatric to adult healthcare underscoring the need for intervention in this age group.
  - Design: Participants randomized to the wait list control arm will be offered a chance to participate in the intervention after the study period is over.
  - Outcomes: the study will assess feasibility outcomes including recruitment rates, withdrawal rates, questionnaire completion, patient engagement and satisfaction, and intervention fidelity as well as efficacy outcomes on JIA self-management, pain interference, self-efficacy, and quality of life. Primary analyses will focus on feasibility outcomes which are clear and well described.
  - Outcomes: The semi-structured interviews of the self-management program participants will provide important information on the acceptability of the program and inform the larger future RCT.
  - Subgroup analyses: while the study will not be powered to find significant interaction effects, this analysis will shed light on important covariates and inform the randomization schema (stratification variables) for the future larger trial.

  Investigators:
  - The research team has extensive experience in this area, both in pediatric rheumatology and in development and implementation of self-management programs. The multidisciplinary research team is led by Dr. Schmeling, a Pediatric Rheumatologist at the University of Calgary/Alberta Children's Hospital. Dr. Schmeling has extensive experience working with patients and researching juvenile idiopathic arthritis (JIA), including in adolescent self-management programs and is the current chair of the Canadian Alliance of Pediatric Rheumatology Investigators (CAPRI). The team has worked together in the first two phases of the project to develop and preliminarily test the self-management program.
  - Consumers: The project includes two consumers, young adults with childhood arthritis, who will be involved in all aspects of the project from a patient perspective. The team also includes a partnership with Cassie & Friends, a charity for children with juvenile arthritis and other rheumatic diseases. Patient input on this project is impressive.

Environment:
- Including five centres across Canada with rural, urban, and ethnically diverse locations will improve generalizability.

Scientific Merit Weaknesses

- Research strategy:
  - The plan is for 4-6 participants to be in the same intervention group. It is expected that 5 patients per month will be recruited. Does that mean then that some patients may have to wait 2 months from when they are randomized until there are enough patients accumulated to form the SMP group of 4-6 participants? This is not discussed explicitly and I wonder about the effects on attrition/adherence. Baseline measures will be collected at T0, prior to randomization. So is it the case that some participants will fill out baseline measures weeks before starting the intervention?
  - (minor) Innovation: The distinction between this proposed program and co-application Dr. Stinson's iPeer2Peer program could have been made more clear. The thought is a group-based intervention might offer some benefits not achieved in the individually matched peer support program
  - Statistical methods: (minor weakness as the effectiveness outcomes are not the primary focus of this RCT) Analyses will need to account for the clustered nature of the data – 4-6 participants will be in the same intervention group so the data will not be independent (you might expect patients in the same group to have outcomes more similar to eachother than patients in different groups).

  Investigators (minor):
  - The research plan notes that Dr. Schmeling has expertise in multi-centre RCTs, and indeed it appears that she has collaborated with co-application Dr. Guzman (PI) on a number of trials in the area, but it does not appear she has been PI of an RCT. The application notes specifically that she will "oversee every aspect of this study;" it is unclear if Dr. Schmeling has done this before. This is a very minor weakness as Dr. Schmeling clearly has clinical expertise and has done research in the area, and her collaborators have RCT experience as PI.

Comments on relevance to Arthritis Society Canada's priority areas

If blank, comments were not provided

-

Comments on Sex & Gender Considerations

Reviewers assessed whether sex & gender were appropriately addressed for this project. Comments, if any, can be found below.

-

Comments on Consumer Involvement

Reviewers were asked to comment if consumers have been included in this project, to what extent, and, if not, is their absence adequately justified. If blank, comments were not provided.

-

Overall Impression

- This application proposes a pilot RCT to evaluate the feasibility and preliminary effectiveness of a virtual group based self-management program in adolescents with juvenile idiopathic arthritis (JIA). This builds on previous work which developed and tested this intervention. The multidisciplinary research team is well suited to carry out the work, and the involvement of consumers and advocates is impressive. The design is reasonable and appropriate for a pilot RCT; two particularly encouraging components are the open label phase – participants randomized to the wait list will get a chance to receive the intervention after the trial is over, and the structured interviews planned to obtain feedback from study participants. Minor concerns include 1) the proposed timeline/amount of time patients might spend waiting for a mentoring group to have sufficient sample size of 4-6; 2) Dr. Schmeling's experience as PI of an RCT; 3) the potential overlap with Dr. Dr. Stinson's iPeer2Peer program.

Recommended Budget

If blank, no comments were provided

-

Secondary Reviewer Feedback

Scientific Merit Strengths

- Need for the program is justified and builds upon previous studies conducted by this group, the proposal is well written, proposed methodology (RCT) is appropriate to study the feasibility. The study will involve existing network (multicentre) which is increase chances of success.

Scientific Merit Weaknesses

- - Limited consideration of gender: it is unclear if the content will include aspects that are relevant to male and female teenagers. Some groups may prefer to be same gender

---

Comments on relevance to Arthritis Society Canada's priority areas

If blank, comments were not provided

-

Comments on Sex & Gender Considerations

Reviewers assessed whether sex & gender were appropriately addressed for this project. Comments, if any, can be found below.

- - Limited consideration of gender: it is unclear if the content will include aspects that are relevant to male and female teenagers. Some groups may prefer to be same gender

---

Comments on Consumer Involvement

Reviewers were asked to comment if consumers have been included in this project, to what extent, and, if not, is their absence adequately justified. If blank, comments were not provided.

-

Overall Impression

- Well written proposal. Important topic. Excellent pilot data. Robust methodology. Excellent team.

Recommended Budget

If blank, no comments were provided

-

External Reviewer Feedback

Scientific Merit Strengths

- Team has previously worked together successfully and brings all necessary expertise plus mult-site/-province settings

  They conducted considerable foundational work leading to this proposal
  Addresses multiple AS priority areas: arthritis pain, IA, childhood (juvenile inflammatory arthritis)

  Considerable foundational work to develop the intervention: Have followed a staged approach to intervention development; Informed by prior systematic review; Only 2 web sites in Canada that provided self-management education and peer mentoring for adolescents with JIA, both led by co-applicant Stinson, both evaluated in pilot studies; Preliminary iteration based on Lorig's self-management theory; Content informed by interviews/focus groups with JIA patients and pediatric rheumatology health professionals; Evaluated acceptability of content/format through focus groups with JIA patients and health professionals; They will develop an intervention manual and train the facilitators

  Methodological details well-described and they will conduct sub-group analysis by demographic characteristics that will consider sex and disease sub-types

Scientific Merit Weaknesses

- Team

  The application states that zero trainees will be involved, and there is no description in the proposal of what trainees will do, but the budget includes $30K for a PhD student and $6K for an undergraduate or graduate student. These are annual expenses.

  Sample size unclear

  Is 4-6 participants across 5 sites enough of a sample size (stated in Summary/Abstract) but later they propose to recruit a total of 100 participants across 5 sites in Calgary, Edmonton, Winnipeg, Vancouver, Toronto. State that formal sample size calculation may not be appropriate for the current phase so they will conduct 2 runs (***unclear***) with 4-6 participants per group at each of 5 sites, thus requiring 50 participants and 50 control.

  Sex-gender

  Also sex/gender mentioned, little attention to diversity; for example, Indigenous or immigrant children. Sampling for diversity would enhance understanding of the feasibility of the intervention prior to more rigorous testing and ultimately the transferability of the intervention to the real world

  Consumer advisors

  The consumer contingent has increased from one at the LOI stage to two at the full application stage. Although PI notes that Cassie & Friends will help to identify additional consumers at each site, we have no way of knowing if that will take place, and it means that only two consumers were potentially involved in developing this proposal (how remains unclear). Having more consumers with diverse characteristics is one way to address SGBA+ and they should be involved from the outset.

  KT plan

  Despite KT plan saying they intend to used integrated and end-of-grant KT, the KT section describes only end-of-grant traditional types of dissemination activities. The end-of-grant dissemination activities are clearly described. However, there is no integrated KT plan, so it’s unclear how the two consumer partners were involved to date and how they will be actively involved throughout the study. Also unclear how partners involved other than in disseminating the results; for example, Cassie & Friends and Canadian Alliance of Pediatric Rheumatology Investigators

  Data analysis

Both qual and quant data collected – no description of how all that data will be integrated to generate a holistic evaluation, which is an important methodological aspect of doing multiple-/mixed-methods research

Feasibility
Did not fully or clearly address concern about potentially limited Internet connectivity other than “we intend to plan for contingencies, such as sending participants pre-recorded sessions and scheduling a follow-up call to address any questions or concerns”, which does not really address the issue

---

Comments on relevance to Arthritis Society Canada's priority areas

If blank, comments were not provided

-

Comments on Sex & Gender Considerations

Reviewers assessed whether sex & gender were appropriately addressed for this project. Comments, if any, can be found below.

- While the applicant notes that most children with IA are female sex, they did not explore the issue of gender, and did not consider related intersection factors, meaning how income, geographic location, ethno-cultural group, etc. interact with sex and gender with respect to the consumer advisors on the research team or sampling, which reduces feasibility of implementing the intervention in the real world if these factors are not explored in early work such as this pilot test for feasibility

---

Comments on Consumer Involvement

Reviewers were asked to comment if consumers have been included in this project, to what extent, and, if not, is their absence adequately justified. If blank, comments were not provided.

-

Overall Impression

- STRENGTHS
  Team has previously worked together successfully and brings all necessary expertise plus mult-site/-province settings

  They conducted considerable foundational work leading to this proposal
  Addresses multiple AS priority areas: arthritis pain, IA, childhood (juvenile inflammatory arthritis)

  Considerable foundational work to develop the intervention: Have followed a staged approach to intervention development; Informed by prior systematic review; Only 2 web sites in Canada that provided self-management education and peer mentoring for adolescents with JIA, both led by co-applicant Stinson, both evaluated in pilot studies; Preliminary iteration based on Lorig's self-management theory; Content informed by interviews/focus groups with JIA patients and pediatric rheumatology health professionals; Evaluated acceptability of content/format through focus groups with JIA patients and health professionals; They will develop an intervention manual and train the facilitators

  Methodological details well-described and they will conduct sub-group analysis by demographic characteristics that will consider sex and disease sub-types

  LIMITATIONS
  Team
  The application states that zero trainees will be involved, and there is no description in the proposal of what trainees will do, but the budget includes $30K for a PhD student and $6K for an undergraduate or graduate student. These are annual expenses.

  Sample size unclear
  Is 4-6 participants across 5 sites enough of a sample size (stated in Summary/Abstract) but later they propose to recruit a total of 100 participants across 5 sites in Calgary, Edmonton, Winnipeg, Vancouver, Toronto. State that formal sample size calculation may not be appropriate for the current phase so they will conduct 2 runs (***unclear***) with 4-6 participants per group at each of 5 sites, thus requiring 50 participants and 50 control.

  Sex-gender
  Also sex/gender mentioned, little attention to diversity; for example, Indigenous or immigrant children. Sampling for diversity would enhance understanding of the feasibility of the intervention prior to more

rigorous testing and ultimately the transferability of the intervention to the real world

Consumer advisors

The consumer contingent has increased from one at the LOI stage to two at the full application stage. Although PI notes that Cassie & Friends will help to identify additional consumers at each site, we have no way of knowing if that will take place, and it means that only two consumers were potentially involved in developing this proposal (how remains unclear). Having more consumers with diverse characteristics is one way to address SGBA+ and they should be involved from the outset.

KT plan

Despite KT plan saying they intend to used integrated and end-of-grant KT, the KT section describes only end-of-grant traditional types of dissemination activities. The end-of-grant dissemination activities are clearly described. However, there is no integrated KT plan, so it’s unclear how the two consumer partners were involved to date and how they will be actively involved throughout the study. Also unclear how partners involved other than in disseminating the results; for example, Cassie & Friends and Canadian Alliance of Pediatric Rheumatology Investigators

Data analysis

Both qual and quant data collected – no description of how all that data will be integrated to generate a holistic evaluation, which is an important methodological aspect of doing multiple-/mixed-methods research

Feasibility

Did not fully or clearly address concern about potentially limited Internet connectivity other than “we intend to plan for contingencies, such as sending participants pre-recorded sessions and scheduling a follow-up call to address any questions or concerns”, which does not really address the issue

---

Recommended Budget

If blank, no comments were provided

-

Consumer Reviewer Feedback

Consumers play an important role in our peer review process. It is important that applicants describe their research in lay language in their submissions. Please note their comments below.

The lay summary and elevator pitch provide a simple overview of the proposed research.

Yes / No:

- Yes

The lay summary describes how the project fits into previous research and their research plan.

Yes / No:

- Yes

The lay summary addresses how the proposal will improve health outcomes and an improved quality of life for people living with arthritis.

Yes / No:

- Somewhat

Are there any feasibility issues that you can identify from your perspective?

Yes / No:

- No

Consumer Comments

- Think this is well thought out and planned.
  I feel that something like this will lead to better disease management for younger patients and will help them to lead more fulfilling lives. Having a chronic illness can be very debilitating and depressing. This should help young people see that there is still so much they can do along with friends.

Comments on relevance to Arthritis Society Canada's priority areas

If blank, comments were not provided

- Work here refers to school.

Comments on Consumer Involvement

Reviewers were asked to comment if consumers have been included in this project, to what extent, and, if not, is their absence adequately justified. If blank, comments were not provided.

-

Overall Impression

- I think this is well thought out and the questionnaires are very relevant to young people. I was also pleased to see that as well as including both genders that there was accommodation for "gender they identify with". I feel this could result in a much better long-term outcome for these clients once they reach adulthood. It is important to teach young people to be their own health advocates as soon as possible, initially with parental involvement, but leading to more personal control..

Recommended Budget

If blank, no comments were provided

-

Scientific Officer Report

The Scientific Officers report captures the spirit of the panel discussion.

Strengths

- - This work builds on previous work completed- solid foundation
  - Multidisciplinary research team
  - PI has extensive research experience in the subject area
  - Collaborators are experts in pain research, PROs in children with chronic diseases, paediatric clinical trials
  - Structured interviews will provide additional data
  - Accommodation for stratification into gender groups as identified by participants

Weaknesses

- - Recruitment timeline not discussed in the application- more clarity on group formation and timeline would help
  - Nationwide study may be difficult to carryout in paediatric patients
  - Are there any interventions tailored to the different gender groups? any anticipated complications based on gender?
  - Diversity of participants groups (immigrants, indigenous) can be improved on
  - Potential exclusion of groups with no internet access
  - Sufficiency of sample size and how does the researcher plan to integrate quantitative and qualitative data

Consumer Feedback

- - Involvement of consumers and advocates is impressive
  - Diversity and internet access disparity are moderate concerns

Overall Impression / Panel Suggestions

- This pilot study would be a good opportunity to assess what percent of the participants are excluded because of lack of internet versus what percentage of participants are able to participate because of internet access (who would otherwise be unable to attend in person sessions).

Recommended Budget

- Budget as requested
